# The Roles of Myeloid-Derived Suppressor Cells in Liver Disease

**DOI:** 10.3390/biomedicines12020299

**Published:** 2024-01-27

**Authors:** Chunye Zhang, Yuxiang Sui, Shuai Liu, Ming Yang

**Affiliations:** 1 Christopher S. Bond Life Sciences Center, University of Missouri, Columbia, MO 65212, USA; zhangcherryuniversity@gmail.com; 2 School of Life Science, Shanxi Normal University, Linfen 041004, China; 3 The First Affiliated Hospital, Zhejiang University, Hangzhou 310006, China; 4 Department of Surgery, University of Missouri, Columbia, MO 65212, USA; 5 NextGen Precision Health Institute, University of Missouri, Columbia, MO 65212, USA

**Keywords:** myeloid-derived suppressor cells, liver inflammation, fibrosis, hepatocellular carcinoma, cell–cell interaction, clinical trials

## Abstract

Liver disease-related mortality is a major cause of death worldwide. Hepatic innate and adaptive immune cells play diverse roles in liver homeostasis and disease. Myeloid-derived suppressor cells (MDSCs) are a heterogeneous population of immature myeloid cells. MDSCs can be broadly divided into monocytic MDSCs and polymorphonuclear or granulocytic MDSCs, and they functionally interact with both liver parenchymal and nonparenchymal cells, such as hepatocytes and regulatory T cells, to impact liver disease progression. The infiltration and activation of MDSCs in liver disease can be regulated by inflammatory chemokines and cytokines, tumor-associated fibroblasts, epigenetic regulation factors, and gut microbiota during liver injury and cancer. Given the pivotal roles of MDSCs in advanced liver diseases, they can be targeted to treat primary and metastatic liver cancer, liver generation, alcoholic and nonalcoholic liver disease, and autoimmune hepatitis. Currently, several treatments such as the antioxidant and anti-inflammatory agent berberine are under preclinical and clinical investigation to evaluate their therapeutic efficacy on liver disease and their effect on MDSC infiltration and function. Phenotypic alteration of MDSCs in different liver diseases that are in a model-dependent manner and lack special markers for distinct MDSCs are challenges for targeting MDSCs to treat liver disease. Multi-omics study is an option to uncover the features of disease-specific MDSCs and potential gene or protein targets for liver disease treatment. In summary, MDSCs play important roles in the pathogenesis and progression of liver disease by regulating both intrahepatic innate and adaptive immune responses.

## 1. Introduction

Liver disease-related mortality is a major cause of death in patients with different liver diseases, such as metabolic dysfunction-associated steatotic liver disease (MASLD), alcoholic liver disease (ALD), and chronic hepatitis [1,2]. Globally, there are about 2 million deaths caused by liver disease in a single year, which are mainly driven by late-stage liver diseases, such as liver cirrhosis and hepatocellular carcinoma (HCC) [3,4]. MASLD, ALD, and chronic hepatitis are the most common types of chronic liver disease that can progress to liver cirrhosis and HCC [5,6,7].

Liver-resident immune cells and infiltrated immune cells during liver disease play essential roles in the maintenance of liver homeostasis, resolution of liver injury, and clearance of pathogens [8,9,10]. In healthy livers, immune cells account for about 14% of total liver cells [11], mainly including macrophages, monocytes, dendritic cells (DCs), neutrophils, natural killer (NK) cells, natural killer T (NKT) cells, myeloid-derived suppressor cells (MDSCs), and B and T lymphocytes [12]. The frequencies of different immune cells change in different liver diseases [13]. Single-cell RNA transcriptome analysis of liver resident cells reveals that the population of each cell type and its expressing gene markers are altered in different conditions [14,15,16].

Accumulating evidence shows that MDSCs play important roles in most liver diseases by regulating both innate and adaptive immune responses. For example, MDSCs can exert their immunosuppressive functions by inducing regulatory T cells and suppressing effector T cell functions in HCC [17,18]. In addition, MDSCs can impair the NK cell cytotoxicity against HCC cells and their interferon-gamma (IFN-γ) production through membrane-bound transforming growth factor beta 1 (TGF-β1) [19]. Recruited C-X-C motif chemokine receptor 2 (CXCR2)-expressing MDSCs in the tumor microenvironment, driven by the C-X-C motif chemokine ligand (CXCL)1/2/5 expressed in liver tumor cells, can also suppress IFN-γ secretion in NKT cells through membrane-bound TGF-β [20]. Therefore, treatments that can regulate the accumulation and activation of MDSCs are potential therapies for malignant liver disease [21,22].

In this review, we first summarize the subtype and function of MDSCs in liver disease, followed by a discussion of the factors that mediate the infiltration of MDSCs and their activation. Then, we discuss the interaction of MDSCs with liver parenchymal cells, mainly including hepatocytes and nonparenchymal cells, such as immune cells. In addition, we will review the current progression in the preclinical and clinical studies targeting MDSCs to treat liver disease.

## 2. The Classification and Markers of MDSCs in Mouse and Human Livers

**Figure 1:**
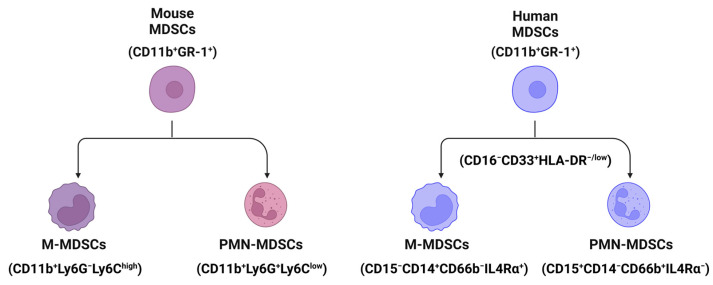
The classification and markers of MDSCs in mouse and human liver tissues. Broadly, MDSCs can be divided into two populations, monocytic MDSCs (M-MDSCs) and polymorphonuclear or granulocytic MDSCs (PMN- or G-MDSCs) using markers shown in the figure. All cartoons in this figure were prepared using Biorender (https://biorender.com, accessed on 26 November 2023).

MDSCs are a heterogenous population of immature myeloid cells [23]. In mice, MDSCs (CD11b^+^GR-1^+^ cells) are broadly divided into two subpopulations (Figure 1): monocytic MDSCs (M-MDSCs, CD11b^+^Ly6G^−^Ly6C^high^ cells) and polymorphonuclear or granulocytic MDSCs (PMN- or G-MDSCs, CD11b^+^Ly6G^+^Ly6C^low^ cells) [24]. In humans, MDSCs (CD11b^+^CD33^+^HLA-DR^−^Lin^−^) can also be further divided into two populations using biomarkers of CD15, CD14, CD66b, and interleukin/IL-4Rα [25]: M-MDSCs (CD15^−^CD14^+^CD66b^−^IL-4Rα^+^) and PMN-MDSCs (CD15^+^CD14^−^CD66b^+^IL-4Rα^−^) (Figure 1). In addition, both MDSCs in humans are CD16^−^CD33^+^HLA^−/low^ cells [26].

The plasticity and differentiation patterns of MDSCs are dependent on disease conditions or environment [27]. Early-stage MDSCs (e-MDSCs, HLA-DR^−^CD33^dim^CD66b^−^ cells) lacking macrophage and granulocyte markers have been shown to accumulate in several diseases [28,29], such as hepatitis B virus (HBV) infection and cardiovascular disease. In addition, single-cell RNA sequencing data indicate that specific subtypes of MDSCs are shown in liver diseases [30,31]. For example, low-density lipoprotein receptor (LDLR)-expressing MDSCs are defined in liver transplantation tissues [30], and these MDSCs highly express genes *TMEM176B* (transmembrane protein 176B), *S100A8* (S100 calcium-binding protein A8), and *S100A9*.

## 3. Pathogenesis of MDSCs in Liver Disease

MDSCs play a pivotal role in different stages of liver disease, from liver inflammation, cell death, and fibrosis to hepatocarcinogenesis. In this section, we review the roles of MDSCs in the pathogenesis of liver disease.

### 3.1. MDSCs in Liver Inflammation

Liver inflammation is a major trigger of liver tissue injury, which can accelerate the development of liver fibrosis and cirrhosis and their progression to primary liver cancer [32,33]. Various etiologies can cause acute and chronic liver inflammation, such as pathogenic microbial infection (e.g., hepatitis virus) [34,35], intake of high-fat and high-sugar diet [36,37], alcohol consumption [38], and toxins. In 2020, liver cancer was the fifth leading cause of cancer death in the United States [39]. Liver inflammation impacts the efficacy of immunotherapies for primary liver cancer, including both HCC and cholangiocarcinoma (CCA).

MDSCs play an essential role in liver inflammation. One study revealed that side scatter (SSC)^high^CD11b^high^Ly-6C^high^Ly-6G^low^ monocytic cells, but not other CD11b^+^Gr-1^+^ MDSCs, can suppress CD4^+^ T cell response by producing nitric oxide (NO). In addition, adoptive transfer of these monocytic MDSCs can significantly decrease concanavalin A (Con A)-induced acute hepatitis in mice [40]. During hepatic ischemia/reperfusion (I/R) injury in mice, accumulation of CD11b^+^Ly-6C^high^ monocytes (M-MDSCs) recruited by the C-C motif chemokine ligand 2 (CCL2)/C-C chemokine receptor 2 (CCR2) axis accelerates liver inflammation, which can be suppressed by CCR2 inhibitor RS504393 and depletion of CCL2 or CCR2 [41]. In addition, the populations of CD11b^+^Ly6G^high^Ly6C^int^ G-MDSCs [42], CD11b^+^Gr-1^+^ MDSCs [43], and monocytic SSC^low^CD11b^+^Gr-1^dim^ MDSCs [44] have been found to be associated with alcoholic or nonalcoholic liver inflammation.

### 3.2. MDSCs in Hepatic Cell Death

Hepatic cell death happens in all different acute and chronic liver diseases with different types of cell death models [45,46], such as cell apoptosis, pyroptosis, ferroptosis, necrosis, and necroptosis. Bone marrow-derived MDSCs induced by the granulocyte-macrophage colony-stimulating factor (GM-CSF) after the stimulation of tumor necrosis factor-alpha (TNF-α) and lipopolysaccharide (LPS) display a protective effect against a lethal dose of acetaminophen (APAP)-induced liver failure by reducing liver infiltration of elastase-expressing neutrophils and inducing apoptosis of activated neutrophils [47].

### 3.3. MDSCs in Liver Fibrosis and Cirrhosis

Bone marrow cells including CD11^+^Gr-1^high^F4/80^−^ cells and CD11^+^Gr-1^high^F4/80^+^ cells can suppress the expression of collagen and α-smooth muscle actin in activated hepatic stellate cells (HSCs) in vitro and in vivo [48]. Accumulation of M-MDSCs (CD11b^+^Ly6G^−^Ly6C^+^ cells) in the livers of mice undergoing bile-duct ligation can inhibit the development of liver fibrosis [49]. The number of granulocytic MDSCs (G-MDSCs) has been shown to be increased in the livers of patients with alcoholic liver cirrhosis (ALC), which is positively correlated with the number of G-MDSCs in peripheral blood [50]. Mechanistically, G-MDSCs not only can increase the plasma levels of arginase I in ALC patients but also can inhibit NK cell-mediated apoptosis of HSCs, resulting in the progression of liver injury and cirrhosis [50].

### 3.4. MDSCs in Hepatocarcinogenesis

In mice with fatty liver and graft injury, arachidonic acid can activate nucleotide-binding oligomerization domain-like receptor family pyrin domain-containing 3 (NLRP3) inflammasome in MDSCs through fatty acid transport protein 2 (FATP2), which can increase IL-17 production in CD4^+^ T cells to cause tumor recurrence [51]. Accumulation of Toll-like receptor 4 (TLR4)-positive monocytic MDSCs in liver graft, which is driven by CXCL10-mediated mobilization, can increase the incidence of HCC recurrence after transplantation. In contrast, HCC recurrence can be suppressed by knocking down or suppressing the CXCL10 or TLR4 signaling pathways [52]. Another study shows that insufficient radiofrequency ablation (RFA) can cause an immunosuppressive microenvironment by upregulating the expression of methyltransferase 1 and significantly increasing the accumulation of PMN-MDSCs or G-MDSCs and TGF-β2 expression to decrease CD8^+^ T cells, resulting in HCC recurrence and progression [18].

Furthermore, the frequencies of total MDSCs and M-MDSCs have been shown to be increased in patients with advanced-stage hepatitis C virus (HCV)-related HCC compared to subjects with early-stage HCC and are positively associated with liver injury and viral load but negatively correlated with the frequency of CD8^+^ T cells [53]. Another study also reveals that lectin-type oxidized LDL receptor-1 (LOX-1)^+^CD15^+^ PMN-MDSCs, which can suppress T cell proliferation and IFN-γ production by producing reactive oxygen species (ROS) and arginase 1 [54], are negatively associated with the overall survival of HCC patients.

Overall, MDSCs play diverse roles at different stages of liver disease through regulation of T cell response and IFN-γ production, antifibrotic function, and inhibition of liver cancer initiation and progression (Table 1).

## 4. The Interactions of MDSCs with Liver Parenchymal and Nonparenchymal Cells

Both liver parenchymal cells including hepatocytes and cholangiocytes and nonparenchymal cells including liver sinusoidal endothelial cells (LSECs), HSCs, Kupffer cells, and different types of lymphocytes can interact with MDSCs through diverse molecules, contributing to important roles in the pathogenesis of liver disease. In this section, we briefly introduce how different liver cells interact with MDSCs to regulate their infiltration, phenotype, and function.

### 4.1. Interaction with Parenchymal Cells

The accumulation of MDSCs in liver injury or cancer is driven by the chemokines/cytokines and their receptors. MDSCs express several chemokine receptors such as CCR2, CXCR2, CXCR4, and CXCR5, while liver tumor cells or malignant hepatocytes express chemokines such as CCL2, CCL5, CXCL1, CXCL5, and CXCL12, and the chemokine/its receptor axis mediates MDSC infiltration in the tumor microenvironment [55,56,57]. The upregulation of hepatic expression of CXCL1 and S100A9 protects fulminant hepatitis by inducing MDSC accumulation [58]. The function and infiltration of MDSCs can be changed in different HCC models, such as a diethylnitrosamine-induced HCC model and a subcutaneous tumor model induced by injection of tumor cells [59]. Cytokines such as granulocyte-colony stimulating factor (G-CSF) and GM-CSF secreted from tumor cells can activate MDSCs to express vascular endothelial growth factor (VEGF) and immunosuppressive factors, resulting in angiogenesis and suppression of immune cells [60].

### 4.2. Interaction with Nonparenchymal Cells

In addition to hepatocytes, LSECs and HSCs can also express CXCL12 to attract the infiltration of MDSCs to the liver tumor microenvironment [56,61]. Activation of MDSCs induced by HSC-condition medium can suppress CD4^+^ and CD8^+^ T cell proliferation by upregulating the gene expression of inducible nitric oxide synthase (iNOS), arginase 1 (Arg-1), and IL-4Rα [61]. The interaction of HSCs with MDSCs is mediated by the molecular-binding prostaglandin E_2_ (PGE_2_) and its receptor 4 (EP4), which specifically induce the subset differentiation of G-MDSC [61]. Accumulation of tumor-infiltrating MDSCs including both G-MDSCs and M-MDSCs can also be regulated by chemokine CX3CL1 in HCC, which is upregulated by adoptive transfer of cytokine-induced killer (CIK) cells, a mixture of immune cells. These MDSCs can suppress the tumor-killing activity of CIKs in HCC in an Arg-1/iNOS-dependent manner, which is reversed by treatment with the phosphodiesterase 5 (PDE5) inhibitor tadalafil [62]. Tumor stromal cells can also induce infiltration of MDSCs by secreting CCL2 and CXCL12 [63]. In addition, MDSC can interact with Kupffer cells to increase their expression of programmed cell death ligand 1 (PD-L1) to induce an immunosuppressive microenvironment. Moreover, the immunosuppressive function of MDSCs is also mediated by inducing the differentiation of regulatory CD25^+^Foxp3^+^CD4^+^ T cells from cocultured CD4^+^ T cells through induction of IL-10 [17].

Furthermore, hepatitis infection regulates MDSC infiltration to suppress the antiviral function of immune cells. For example, CD33^+^CD11b^low^HLA-DR^low^ MDSCs stimulated by HCV can impair the antiviral ability of NK cells by reducing their IFN-γ production [64]. MDSC-mediated suppression of NK cell function is mediated by the production of arginase 1, which is independent of cell–cell interaction.

## 5. Factors That Impact MDSC Infiltration and Function during Liver Injury

Given the aforementioned functions of MDSCs in liver disease, it is critically important to investigate the key factors that impact MDSC infiltration and function in liver pathogenesis. These factors can be regulated or targeted to develop MDSC-mediated therapies for liver disease. Here, we review some key factors that regulate the recruitment of MDSCs and their functions.

### 5.1. Inflammation

**Figure 2:**
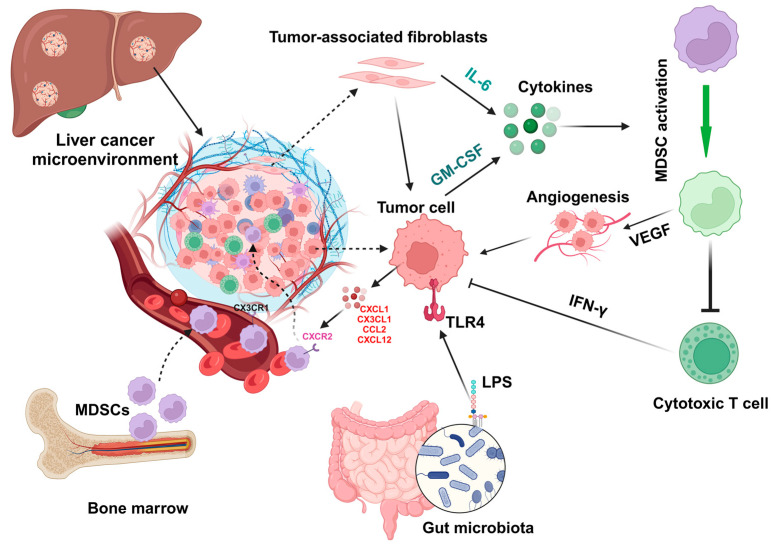
Factors regulate the activation and infiltration of MDSCs in liver cancer. During liver injury and hepatocarcinoma, chemokines such as CXCL1, CX3CL1, CCL2, and CXCL12 and cytokines such as IL-6 and GM-CSF can be expressed by tumor-associated fibroblasts and tumor cells to regulate the infiltration and activation of MDSCs, which can promote angiogenesis by expressing express vascular endothelial growth factor (VEGF) to inhibit the function (e.g., IFN-γ production) of cytotoxic T cells. In addition, gut microbiota-derived components such as lipopolysaccharides (LPSs) can activate hepatocytes or tumor cells by interacting with Toll-like receptor 4 (TLR4) to upregulate CXCL1 expression, resulting in the migration of MDSCs into the microenvironment. All cartoons in this figure were prepared using Biorender (https://biorender.com, accessed on 29 November 2023).

Proinflammatory cytokine IL-1β can induce overexpression of solute carrier family 7 member 11 (SLC7A11) in HCC cells to enhance tumor metastasis. The upregulation of SLC7A11 induces the infiltration of tumor-associated macrophages (TAMs) and MDSCs by activating the colony-stimulating factor 1 (CSF1)/colony-stimulating factor 1 receptor (CSF1R) axis [65]. Inflammatory mediators such as CX3CL1 and IL-13 in the HCC tumor microenvironment can regulate the infiltration of MDSCs (Figure 2) that contribute to the immunosuppressive function of cytokine-induced killer cells [62].

### 5.2. Chemokines and Cytokines

High levels of baseline IL-6 in patients with unresectable HCC have been associated with poor response rates to the treatment of atezolizumab and bevacizumab and low overall survival [66]. Cytokines expressed by tumor cells or endothelial cells in the tumor microenvironment, such as GM-CSF and IL-6 (Figure 2), can promote MDSC induction to suppress antitumor IFN-γ^+^ T cell production and increase angiogenesis in the mouse HCC microenvironment [67]. Neutralization of GM-CSF and IL-6 can decrease the accumulation of MDSCs to suppress HCC progression. HCC progression and MDSC accumulation are also abrogated in chemerin (retinoic acid receptor responder protein 2)-deficient mice, indicating the protective role of chemerin against HCC. In addition, the circulating concentrations of GM-CSF or IL-6 are positively associated with the infiltration of tumor-infiltrating MDSCs, as well as the levels of chemerin in the tumor, in patients with HCC [67].

### 5.3. Tumor-Associated Fibroblasts

In human HCC, M-MDSCs are enriched in the fibrotic livers surrounding the tumor area, and the expression of M-MDSC marker CD33 is positively associated with tumor progression and negatively associated with the survival rate of HCC patients [68]. In mouse HCC models, M-MDSC enrichment in fibrotic livers increases tumor development, which is associated with the reduction in tumor-infiltrating lymphocytes. The increase in M-MDSCs in the fibrotic liver is triggered by activated HSCs through p38 mitogen-activated protein kinase (MAPK) signaling, which can be suppressed to inhibit the crosstalk between HSCs and M-MDSCs to result in the suppression of HCC growth [68].

In the HCC tumor microenvironment, chemokines and cytokines, such as stromal cell-derived factor 1 alpha (SDF-1α, CXCL12a) and IL-6, can induce MDSC infiltration and activation (IL-6/STAT3-mediated) to suppress the antitumor immune response and promote tumor progression [69]. IL-6 secreted from HSCs, the major cells transdifferentiated into myofibroblasts in the liver after activation, can induce the production of MDSCs from bone marrow cells and activate the expression of iNOS and Arg-1. In addition, HSC-treated MDSCs increased their inhibitory function in the T cell immune response in the tumor microenvironment [70].

### 5.4. Epigenetic Regulation

Epigenetic regulation, such as DNA methylation, histone modification, and transcription by noncoding RNAs, influences liver physiology and pathology and impacts liver disease development [71,72]. The increased expression of PHD finger protein 19 (PHF19), an epigenetic regulator, predicts poor prognosis in patients with HCC. Mechanistically, PHF19 regulates the cell cycle and DNA replication, and high PHF19 expression is positively associated with the infiltration of MDSCs and Th2 helper T cells [73].

### 5.5. Gut Microbiota

The gut microbial components lipopolysaccharides (LPSs) can activate TLR4, a family member of pattern recognition receptors (PRRs), on HCC cells to regulate nuclear factor-κB (NF-κB) and MAPK signaling pathways, resulting in cancer cell proliferation [74]. Activation of the NF-κB signaling pathway can also promote the invasion of HCC cells by regulating extracellular matrix (ECM) remodeling, the expression of degradation enzyme matrix metalloproteinases (MMPs), and epithelial–mesenchymal transition (EMT), as well as angiogenesis in the tumor microenvironment [75]. In addition, overexpression of NF-κB can increase the resistance of HCC cells to chemotherapy and radiotherapy [76,77].

Berberine, a herbal isoquinoline alkaloid compound with antioxidant and anti-inflammatory activities [78,79], can reduce alcoholic hepatic injury in mice by activating G-MDSC-like cells through activation of the IL-6/signal transducer and transcription 3 (STAT3) signaling pathway and regulation of the gut microbial profile with an increase in the abundance of *Akkermansia muciniphila*. In contrast, an antibiotic cocktail treatment causes depletion of gut microbiota and reduces the population of G-MDSCs in the liver, resulting in the abrogation of the protective effect of berberine against alcohol-induced liver injury [80]. Meanwhile, studies also show that oral supplementation of *A. muciniphila* can reduce alcohol-induced liver injury [81,82]. Treatment of berberine also increases the abundance of *A. muciniphila* in high-fat diet (HFD)-fed mice. In summary, the effect of berberine on G-MDSCs is highly regulated by gut microbial species *A. muciniphila*.

In mice with primary sclerosing cholangitis (PSC) or colitis, a leaking gut increased the presence of gut microbiota and LPS in the liver, which increased the expression of CXCL1 in hepatocytes by activating the TLR4 signaling pathway (Figure 2), resulting in an accumulation of CXCR2-expressing PMN-MDSCs [83]. Gut microbiota dysbiosis in mice lacking the inflammasome sensor molecule NOD-like receptor family pyrin domain-containing 6 (NLRP6) has increased the expansion of M-MDSCs in the liver in a TLR4-dependent manner, resulting in a reduction in T cell population [84]. The supplementation of *A. muciniphila* improves gut barrier function to suppress liver inflammation and fibrosis, which is negatively associated with the abundance of M-MDSCs in the caeca [84].

In addition to the above factors, transcriptional factors play important roles in the immunosuppressive function of MDSCs. For example, treatment with STAT3 inhibitors can suppress the frequency of liver-associated MDSCs to inhibit tumor growth and dampen the suppressive function of MDSCs to enhance the anticancer efficacy of chimeric antigen receptor T (CAR-T) cells [85]. Furthermore, hypoxia in the primary HCC tumor microenvironment can also drive the recruitment of CX3CR1-expressing MDSCs via its ligand CCL26 [86].

## 6. Roles of MDSCs in Different Liver Diseases

Given the varied roles of MDSCs in liver disease, targeting MDSCs to regulate liver immunity is a strategy to treat liver disease [87,88,89], especially for liver cancers in different models. In this section, we review current methods and strategies that regulate MDSC infiltration and function in liver diseases.

### 6.1. Hepatocellular Carcinoma

Anti-liver cancer treatments can regulate the infiltration of MDSCs and their function. In mice with HCC, sorafenib treatment can inhibit HCC growth, which is associated with a decrease in immunosuppressive cells, including both MDSCs and regulatory T cells [90]. Another study shows that the adoptive transfer of MDSCs to HCC-bearing mice not only promotes HCC progression, partially by activating tumor-associated fibroblasts via IL-6/fibroblast growth factor 1 (FGF1) signaling, but also induces resistance to sorafenib treatment [91].

Treatment with 5-fluorouracil (5-FU) can increase the infiltration of MDSCs to suppress the efficacy of anti-PD-L1 antibodies in mice with orthotopic HCC. Mechanistically, VEGF-A expressed by tumor cells through activation of peroxisome proliferator-activated receptor-gamma (PPARγ) stimulates MDSC expansion to suppress CD8^+^ T cell function [92]. Therefore, PPARγ antagonist treatment can resensitize tumor cells to anti-PD-L1 treatment. Similarly, in human HCC, the number of MDSCs increased post-transarterial chemoembolization, which is negatively associated with the number of CD8^+^ T cells [92]. The frequency of PD-L1^+^ MDCSs has also been shown to be significantly increased in the PBMCs of patients with HCC compared to that in healthy subjects, and these cells can secrete high levels of VEGF-A [93]. Therefore, angiogenesis and immunosuppressive factors secreted from MDSCs can inhibit the efficacy of anti-HCC treatments.

A mouse study shows that HCC mice that have less tumor infiltration of MDSCs and regulatory T cells in the tumor are responders to anti-CD137 antibody treatment. In addition, depletion of MDSCs using the anti-mouse Gr-1 antibody significantly improves the survival of tumor-bearing mice [94]. Another study reveals that the blockade of MDSC infiltration in mice with primary HCC or colorectal cancer liver metastasis using CXCR2 inhibitor SB225002 can significantly improve anti-PD-1 immunotherapy and improve the survival rate of HCC-bearing mice [88]. Treatment with chemokine receptor inhibitors impairs the infiltration of MDSCs and tumor-associated macrophages to the tumor microenvironment to abolish their immunosuppressive function against cytotoxic CD8^+^ T cells [88,95].

### 6.2. Cholangiocarcinoma

Depletion of tumor-associated macrophages by the anti-CSF1R (colony-stimulating factor 1 receptor) antibody failed to suppress murine CCA due to a compensatory infiltration of G-MDSCs with immunosuppressive features [96]. In contrast, dual treatments with anti-CSF1R and anti-Ly6G antibodies can significantly improve the efficacy of anti-PD-1 therapy to increase the survival time of CCA mice [96]. Fibroblast activation protein (FAP)-mediated progression of intrahepatic cholangiocarcinoma (ICC) can be abrogated by anti-Gr-1 antibody treatment, as FAP mediates the infiltration of MDSCs in ICC via inducing CCL2 expression to promote tumor progression and angiogenesis [97].

### 6.3. Metastatic Liver Cancer

About 50% of patients with colorectal cancer will develop liver metastases. The frequency of CD14^+^HLA-DR^−/low^ MDSCs has been shown to increase in patients with colorectal cancer metastasis, and these MDSCs contribute to forming the premetastatic niche and are associated with inhibition of T cell proliferation and poor prognosis [98]. Intravascular infection of TLR9 agonist ODN2395 via the portal vein can significantly suppress tumor progression by regulating MDSC depletion and programming in mice with colon adenocarcinoma liver metastasis [99].

### 6.4. Subcutaneous Liver Cancer

Artemisinin (ART), an antimalarial drug with tumoricidal and immunoregulatory properties, can induce MDSC apoptosis and inhibit their accumulation and immunosuppressive function in vitro. In vivo, treatment of ART at doses of 50 mg/kg and 100 mg/kg is able to significantly suppress tumor growth in mice with subcutaneous Hepa 1-6-induced hepatoma by reducing the frequencies of M-MDSCs and G-MDSCs [100].

In mice with subcutaneous xenograft HCC (human liver cancer cell lines such as HepG2, Huh-7, and MHCC97H), curcumin treatment can inhibit the frequency of CD11b^+^GR-1^+^ MDSCs and suppress the expression of G-CSF and GM-CSF by suppressing the TLR4/NF-κB signaling pathway [60].

### 6.5. Liver Regenration

In solid organs of the body, only the liver can regenerate to return to the original ratio of organ-to-bodyweight [101]. In the early stage of liver regeneration, MDSCs have unique transcriptional profiles that increase ROS production and angiogenesis, contributing to liver regeneration [102].

### 6.6. Autoimmune Hepatitis

Liver X receptor alpha (LXRα)-deficient mice have an increased expansion of both PMN-MDSCs and M-MDSCs in the liver compared to wild-type mice, resulting in amelioration of concanavalin A (ConA)-induced hepatitis [103]. Mechanistically, MDSCs from LXRα^−/−^ mice have lower expression of interferon regulatory factor 8 (IRF-8) with increased capabilities of proliferation and survival compared to MDSCs from wild-type mice [103].

### 6.7. Alcoholic and Nonalcoholic Liver Diseases

In addition to hepatitis viral infection, MASLD and ALD are the most common chronic liver diseases that are able to induce liver cancer initiation and progression [104,105]. The population of G-MDSCs (expressing CD11b^+^Ly6G^high^Ly6C^int^) was increased in the blood, spleen, and liver of alcohol-treated mice. G-MDSCs have a protective role at the early stage of alcohol-induced liver injury, as depletion of these cells can increase serum levels of liver injury enzymes alanine aminotransferase and aspartate aminotransferase, while adoptive transfer of G-MDSCs can ameliorate acute alcoholic liver damage [42].

The increased frequency of CD11b^+^Gr-1^+^ MDSCs in peripheral blood and accumulation of Gr-1^+^ cells in the liver are positively associated with MASLD, which can be suppressed by antioxidant treatment (MitoTEMPO) to reduce liver inflammation by suppressing the expression of MDSC-related proinflammatory mediators, such as S100A8 and S100A9 [43]. Another study also shows that monocytic SSC^low^CD11b^+^Gr-1^dim^ MDSCs recruited by the CCL2/CCR2 axis into the liver display a very strong suppressive ability on T cell response by producing NO in mice with MASLD [44].

## 7. Current Clinical Trials of MDSC-Regulated Therapies in Liver Disease

The presence of MDSCs is associated with liver cancer progression in mouse models and human patients with liver cancer [85,89]; therefore, targeting MDSCs is a strategy for liver cancer treatment. Clinically, different treatments (Table 2), such as small or short activating RNAs [22], liver X nuclear receptor (LXR) agonist RGX-104 (not validated in liver cancer) [106], a HepaVac-101 vaccine consisting of multipeptide antigens (IMA970A) plus TLR7/8/RIG-I (retinoic acid-inducible gene I) agonist CV8102 [107], TLR8 agonist (GS-9688) [108], and invariant NKT cells (iNKT) infusion [109], are under investigation in the clinic (https://clinicaltrials.gov/, accessed on 20 November 2023). In addition, surgical resection can also decrease the accumulation of MDSCs in patients with hepatitis B virus-related HCC [110]. However, treatments directly targeting MDSCs are less studied, and more pharmaceutical medicines should be developed to regulate the function and frequency of MDSCs in liver diseases.

## 8. Challenges and Future Directions

MDSCs, a heterogeneous population, mediate both innate and adaptive immune responses in liver homeostasis and injury. They are involved in the pathogenesis of most liver diseases, such as ALD, MASLD, hepatitis, liver fibrosis, cirrhosis, and HCC, by regulating the interaction with both liver parenchymal cells such as hepatocytes and nonparenchymal cells. MDSCs can be broadly divided into two populations: monocytic MDSCs (M-MDSCs) and polymorphonuclear or granulocytic MDSCs (PMN- or G-MDSCs). Hepatic infiltration and activation of MDSCs can be regulated by inflammatory chemokines (e.g., CXCL1 and CCL2) and cytokines (e.g., IL-6), tumor-associated fibroblasts, epigenetic factors, and gut microbiota during liver pathogenesis. Given all these factors can impact the infiltration, phenotype, and function of MDSCs, it is very hard to define a specific subtype of MDSCs in liver diseases. In addition, the population of MDSCs can also be changed in a model-dependent manner. A multi-omics study can be performed in each chronic liver disease to uncover the features of disease-specific MDSCs and potential gene or protein targets for liver disease treatment.

Overall, MDSCs play important roles in the progression of chronic liver disease by regulating both intrahepatic innate and adaptive immune responses. MDSCs are optional targets for the treatment of primary and metastatic liver cancer, liver generation, and autoimmune hepatitis. However, only a few drugs are under evaluation for their therapeutic efficacy and potential synergistic effects with other treatments. Therefore, new medicines or strategies that can regulate the function and migration of MDSCs are needed.

## Figures and Tables

**Table 1:** The functions of MDSCs in liver diseases.

Liver Disease	MDSC Subtypes	MDSC Markers	Function	References
Hepatitis	M-MDSCs	SSC^high^CD11b^high^Ly-6C^high^Ly-6G^low^ MDSCs	Suppressing CD4 T cell response	[40]
Liver I/R injury	M-MDSCs	CD11b^+^Ly-6C^high^ M-MDSCs	Increasing liver inflammation	[41]
Acute alcoholic liver injury	PMN-MDSCs	CD11b^+^Ly6G^high^Ly6C^int^ MDSCs	Protecting alcoholic liver disease in the early stage	[42]
Nonalcoholic liver disease	MDSCs	CD11b^+^Gr-1^+^ MDSCs	Proinflammatory function	[43]
Nonalcoholic liver disease	MDSCs	SSC^low^CD11b^+^Gr-1^dim^ MDSCs	Suppressing T cell response	[44]
Liver failure	MDSCs	LPS-treated MDSCs	Promoting apoptosis of activated neutrophils	[47]
Liver fibrosis	MDSCs	CD11b^+^Gr-1^+^F4/80^+/−^ MDSCs	Antifibrotic function	[48]
Liver fibrosis	M-MDSCs	M-MDSCs or CD11b^+^Ly6G^−^ Ly6C^+^ cells	Antifibrotic function	[49]
Liver cirrhosis	PMN-MDSCs	PMN- or G-MDSCs	Promoting liver cirrhosis	[50]
HCC	M-MDSCs	TLR4^+^ M-MDSCs	Increasing HCC recurrence	[52]
HCC	PMN-MDSCs	PMN-MDSCs	Promoting tumor recurrence	[18]
HCV-related HCC	M-MDSCs	Total MDSCs and M-MDSCs	Correlating with the HCC stage	[53]
HCC	PMN-MDSCs	LOX-1^+^CD15^+^ PMN-MDSCs	Inhibiting T cell proliferation and IFN-γ production	[54]

Abbreviations: HCC: hepatocellular carcinoma; HCV: hepatitis C virus; I/R: ischemia/reperfusion; int: intermediate; LOX-1: lectin-type oxidized LDL receptor-1; MDSCs: myeloid-derived suppressor cells; SSC: side scatter; TLR4: Toll-like receptor 4.

**Table 2:** Clinical trials of MDSC-regulated therapies in liver disease.

Trials	Phase	Treatment	Results and Measurement	References
NCT04105335	1	MTL-CEBPA (small or short activating RNAs)	This treatment can induce HCC regression in patients by significantly decreasing the number of blood monocytic myeloid-derived suppressor cells (M-MDSCs) and protumoral M2 tumor-associated macrophages (TAMs).	[22]
NCT02922764	1	RGX-104	It is an oral small molecule that can suppress tumor-infiltrating MDSCs by targeting the liver X receptor (LXR) in multiple tumor models.	[106]
NCT03188276	1	Ledipasvir/sofosbuvir and daclatasvir/sofosbuvir	The relationship between the antiviral treatment effect and the functional activity of MDSCs and natural killer (NK) cells in chronic hepatitis C will be evaluated.	N/A
NCT03203005	1/2	HepaVac-101 vaccine	The vaccine consists of multipeptide antigens (IMA970A) plus TLR7/8/RIG I agonist CV8102, and its effect on the frequencies of MDSCs will be performed using peripheral blood mononuclear cells (PBMCs).	[107]
NCT03491553 NCT03615066	2	TLR8 agonist GS-9688 (selgantolimod)	GS-9688 treatment can reduce the frequency of regulatory T cells and M-MDSCs, while it can increase TNF-related apoptosis-inducing ligand-expressing NK cells and degranulation of arginase-I-expressing PMN-MDSCs.	[108]
NCT04011033	2/3	Invariant NKT cells (iNKT) infusion	Frequencies of immune cells including MDSCs will be analyzed by flow cytometry before and after iNKT infusion.	[109]
NCT02868255	N/A *	Anti-SIRPα antibody	The study aims to test the expression of signal regulatory protein-α (SIRPα)/CD47 signaling and the efficacy of anti-SIRPα antibodies in patients with HCC and ovarian cancer.	N/A
N/A	N/A	Liver resection	After liver resection surgery, the accumulation of MDSCs in patients with hepatitis B virus-related HCC was decreased.	[110]

* N/A: not applicable.

## Data Availability

All reports supporting the discussion are available in this paper.
